# Probe Microscopic Studies of DNA Molecules on Carbon Nanotubes

**DOI:** 10.3390/nano6100180

**Published:** 2016-10-08

**Authors:** Kazuo Umemura, Katsuki Izumi, Shusuke Oura

**Affiliations:** Biophysics Section, Tokyo University of Science, 1-3 Kagurazaka, Shinjuku, Tokyo 162-8601, Japan; katsu.win2014@gmail.com (K.I.); el_ni_nol0@yahoo.co.jp (S.O.)

**Keywords:** probe microscope, force microscope, tunneling microscope, DNA, carbon nanotube

## Abstract

Hybrids of DNA and carbon nanotubes (CNTs) are promising nanobioconjugates for nanobiosensors, carriers for drug delivery, and other biological applications. In this review, nanoscopic characterization of DNA-CNT hybrids, in particular, characterization by scanning probe microscopy (SPM), is summarized. In many studies, topographical imaging by atomic force microscopy has been performed. However, some researchers have demonstrated advanced SPM operations in order to maximize its unique and valuable functions. Such sophisticated approaches are attractive and will have a significant impact on future studies of DNA-CNT hybrids.

## 1. Introduction

Scanning probe microscopy (SPM) has become one of the most popular methods for the nanoscopic characterization of various materials [1,2,3,4,5,6,7,8,9,10,11]. Scanning tunneling microscopy (STM) was established in 1981 as the eldest brother of the SPM family [12,13]. In STM, a conductive probe and a conductive sample are required for stable observation. When the probe approaches the sample surface with suitable bias voltage, the tunneling current that is affected by the surface density of states of the sample and the distance between the probe and the sample can be detected. By monitoring the tunneling current, it is possible to map the surface density of states by STM. The second eldest brother of the SPM family is atomic force microscopy (AFM), which was established in 1986 [9,14,15]. In AFM, a probe is fabricated on a cantilever. Because the cantilever is a type of plate spring, it is warped when it comes near a sample surface. By monitoring the warping of the cantilever, it is possible to map surface morphology by AFM. In the case of AFM, the probe and sample should not be conductive. For this reason, AFM has been widely employed for nanoscopic characterization of biomolecules, including DNA molecules.

**Figure 1:**
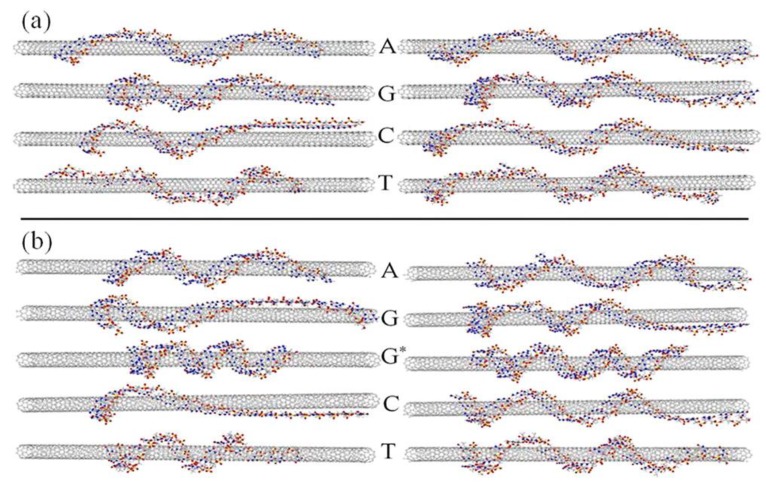
Theoretical structural models of single-stranded DNA (ssDNA)-single-walled nanotube (SWNT), SWNT hybrids with different DNA bases. (**a**,**b**) show (6,5) and (9,1) SWNTs, respectively. Left and right images show 23 and 29 mers of ssDNA, respectively. G* indicates the guanine mer with fixed ends. The most stable structures with the strongest binding energies based on π–π interactions are represented. The model indicated that the wrapping of DNA molecules is not homogeneous, and helical wrapping is not ideal (reprinted from reference [27] with permission).

Hybrids of DNA and carbon nanotubes (CNTs) were reported in 2003 in two independent studies [16,17]. Although CNTs are insoluble in aqueous solutions, hybrids of DNA and CNT (DNA-CNT hybrids) are water-soluble. Furthermore, by wrapping CNT surfaces with DNA molecules, CNTs can be mono-dispersed. The mono-dispersed CNTs reveal superior electrical and optical properties. By using the mono-dispersed DNA-CNT hybrids in aqueous solutions, their use in various biological applications, such as nanobiosensors and drug delivery systems, has become possible [18,19,20,21,22,23,24,25,26]. In addition to experimental research, theoretical structural models of DNA-CNT hybrids have been proposed by several researchers [27,28,29,30,31,32]. A theoretical structural model of hybrids of single-stranded DNA (ssDNA) and single-walled nanotubes (SWNTs), as reported by Michael et al. [27], is indicated in Figure 1. The DNA length, types of DNA sequences, and different adsorption mechanisms differ according to the types of SWNTs.

SPM is a powerful tool for the nanoscopic characterization of DNA-CNT hybrids. Morphological characterization by AFM imaging is the most popular approach; however, SPM reveals much more information in addition to morphology. In fact, after STM and AFM were established, several SPM-related techniques were developed. Using SPM, the physical and chemical properties of DNA-CNT hybrids can be characterized. 

In this review, we discuss nanoscopic studies of DNA-CNT hybrids using various types of SPMs. Our goal is to provide useful information for researchers in various fields who wish to characterize DNA-CNT hybrids.

## 2. Atomic Force Microscopic Observation of DNA-Carbon Nanotube (CNT) Hybrids

The most popular method of using SPM for DNA-CNT hybrids is morphological characterization by AFM imaging. In fact, topographic imaging is the original function of AFM. In initial reports of DNA-CNT hybridization, AFM was used to confirm the mono-dispersion of CNTs with DNA molecules [16,17]. Zheng et al. employed the commercially available AFM with intermittent contact mode in air [17]. In general, there are three scanning modes in AFM imaging: contact mode, intermittent contact mode, and non-contact mode. Contact mode and intermittent contact mode detect repulsive forces between a probe and a sample surface, whereas non-contact mode is employed to measure attractive forces.

In contact mode, static bending of a cantilever with a small spring constant is translated into force values based on Hooke’s law. Thus, a soft cantilever is more sensitive than a hard one. In intermittent contact mode and non-contact mode, a cantilever oscillates with resonance frequency using a piezo and AC voltage. Then, the amplitude shift caused by interactions between the probe and the sample surface is detected. For these modes, hard cantilevers are preferred in order to obtain suitable resonance frequency, as it is difficult to obtain sharp oscillation peaks with a soft cantilever.

Although contact mode is suitable for obtaining high-resolution images on a hard sample surface, the friction force between the probe and the sample surface is not negligible, since scanning is carried out with static bending of the cantilever. If contact mode is employed for DNA-CNT hybrids deposited on a flat substrate surface, the friction force may cause problems for stable imaging, as DNA-CNT hybrids protrude from the substrate surface. The use of non-contact mode is available for DNA-CNT hybrids. However, non-contact mode imaging is difficult in comparison with intermittent contact mode, since weak attractive forces are detected. For these reasons, intermittent contact mode has been employed in most studies.

Another important point to consider is the experimental environment during AFM imaging. In general, three different conditions are available: vacuum, gas, and liquid. For gas and liquid conditions, various types of gases and liquids can be selected.

Although various environments are available for AFM imaging, most studies present AFM images obtained in air. For example, Zheng et al. revealed clear AFM images of mono-dispersed SWNTs wrapped with ssDNA molecules, by separating semi-conductive and conductive SWNTs wrapped with ssDNA by anion exchange chromatography. The elution speed of the conductive SWNTs was faster than that of the semi-conductive SWNTs. Then, lengths of each DNA-CNT hybrid were measured by AFM, and the lengths of semi-conductive and conductive SWNTs were compared. The AFM data confirmed that the average length of the conductive SWNTs was shorter than that of the semi-conductive SWNTs in the samples.

In many studies, AFM imaging is routinely used to characterize DNA-CNT hybrids. We present several examples of AFM imaging of DNA-CNT hybrids.

Quantitative analysis of the length of observed objects is one of the most popular advantages of AFM imaging [33,34,35,36,37,38]. Palma et al. prepared connected SWNT hybrids with DNA molecules including Y-shaped end-to-end SWNT junctions, and then precisely characterized the length of the hybrids by AFM cross-section analysis [33]. In their samples, the average length of the initial DNA-SWNT hybrids was 147.7 ± 92.8 nm. When four hybrids were cross-linked, the average length increased to 418.2 ± 370.1 nm. Asada et al. compared the photoluminescence (PL) spectra and length distribution of DNA-SWNT and DNA-DWNT hybrids in order to prove that the PL spectra are affected not only by the chirality and diameter of SWNTs, but also their length [34]. The prepared hybrids were separated by high-performance liquid chromatography (HPLC) with COSMOSIL CNT columns. Then, several fractions obtained by HPLC were characterized by PL and AFM. The results indicated that every fraction included different average lengths, and the lengths affected the PL intensity. Koh et al. compared the lengths of DNA-SWNT hybrids that were prepared using different types of SWNTs—e.g., produced by arc-discharge (AD) method, by chemical vapor deposition (CVD) method, and by high-pressure carbon monoxide process (HiPco) method [35]. The prepared hybrids were characterized by AFM and near infrared (NIR). The length analysis by AFM, indicated that the lengths differed according to the SWNT types and the sonication conditions. For example, in the case of AD-produced SWNTs, the average lengths were 747, 329, and 189 nm for sonication for 6 h at 3 W, 34 h at 3 W, and 34 h at 77 W, respectively. Khripin et al. verified the lengths of DNA-SWNT hybrids after precipitation with various concentrations of polyethylene glycol (PEG) [36], suggesting that length separation of SWNT is possible using DNA and PEG. After recovering DNA-SWNT hybrids after PEG precipitation, the lengths of the collected hybrids were characterized by AFM. Short and long SWNTs could be precipitated at high and low PEG concentrations, respectively. Yamamoto et al. carefully characterized hybrids of DNA and SWNTs by combining AFM, NIR absorption, and PL. From measurement of length by AFM, it was found that DNA-SWNT hybrids could be separated by column chromatography based on different SWNT lengths [26].

Other important data obtained by cross-section analysis of AFM images are the heights of the observed objects [39,40,41,42,43,44,45,46]. For example, Gladchenko et al. precisely analyzed the heights of SWNTs wrapped with single-stranded polyriboadenylic acids, poly(rA), and polyribocytidylic acids, poly(rC), by cross-section analysis of AFM images [39]. Because AFM images are composed of line scans of a sample surface, the height information of the observed object can be easily obtained. Heights were measured at many positions for one hybrid, and non-uniform adsorption of poly(rA) was observed. Hayashida et al. examined the differences between the heights of ssDNA-SWNT hybrids and double stranded DNA (dsDNA)-SWNT hybrids using CVD and HiPco SWNTs [40]. It was found that un-uniform DNA adsorption appeared in HiPco SWNTs at low DNA concentration. Nii et al. demonstrated the reaction of single-stranded DNA-binding (SSB) proteins with ssDNA-SWNT and dsDNA-SWNT hybrids, and discussed SSB binding using the height analysis of AFM images [41]. Height distribution became broad after the SSB binding to ssDNA-SWNT hybrids. In the case of dsDNA-SWNT hybrids, height distribution was not changed even after addition of SSB proteins. Campbell et al. attached quantum dots to DNA-CNT hybrids, and confirmed quantum dot attachment by height and width analysis based on AFM observations [42]. They succeeded in observing 14nm pitch in helical turns.

Shahrokhian et al. proposed a DNA sensor using glassy carbon electrodes (GCEs) and multi-walled carbon nanotubes (MWNTs). MWNTs on GCEs were modified with ssDNA molecules, and then, complementary ssDNA molecules were hybridized on the modified electrode surface [47]. Although electrochemical measurements were primarily used, AFM imaging of the electrode surfaces was also carried out. For this purpose, single molecules were not observed by AFM imaging. The roughness of the modified electrode surfaces was estimated from AFM images.

**Figure 2:**
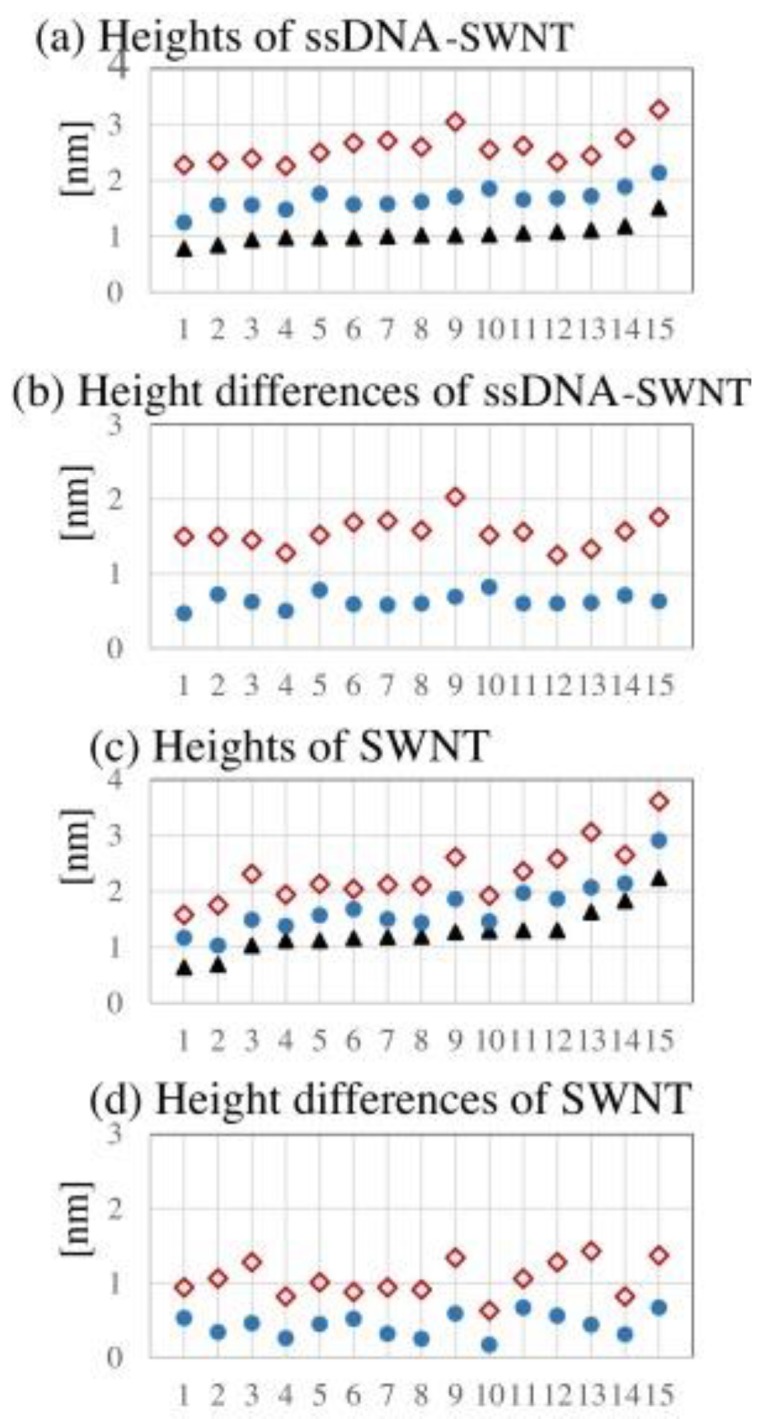
Height values obtained by simultaneous imaging of individual ssDNA-SWNT hybrids and bare SWNT molecules by three different measurement conditions. X-axis indicates No. of individual hybrid. Y-axis indicates heights ((**a**,**c**) and height differences (**b**,**d**)). (**a**) Heights of ssDNA-SWNT hybrids. Red rhombuses: intermittent contact mode in TE buffer. Blue circles: non-contact mode in air. Black triangles: intermittent contact mode in air; (**b**) Subtracted height values of ssDNA-SWNT hybrids between two different atomic force microscopy (AFM) force conditions. Red rhombuses: subtraction between intermittent contact mode in TE buffer and intermittent contact mode in air. Blue circles: subtraction between non-contact mode in air and intermittent contact mode in air; (**c**) Heights of bare SWNTs. Red rhombuses: intermittent contact mode in TE buffer. Blue circles: non-contact mode in air. Black triangles: intermittent contact mode in air; (**d**) Subtracted height values of bare SWNT molecules between two different AFM force conditions. Red rhombuses: subtraction between intermittent contact mode in TE buffer and intermittent contact mode in air. Blue circles: subtraction between non-contact mode in air and intermittent contact mode in air. Maximum heights were obtained with intermittent contact mode in an aqueous solution (reprinted from reference [48] with permission).

Recently, several studies have reported AFM observation of DNA-CNT hybrids in aqueous solutions [48,49]. As expected, much larger values for the heights of DNA-CNT hybrids were obtained in aqueous solutions. Hayashida et al. demonstrated simultaneous imaging of the same hybrids by intermittent contact mode in air, intermittent contact mode in liquid, and non-contact mode in air [48]. When DNA-CNT hybrids were observed by non-contact mode in air, the height of the hybrids was significantly increased in contrast to that by intermittent contact mode in air (Figure 2). Their data indicated that the morphology of DNA-CNT hybrids is strongly affected by the conditions of AFM imaging.

Various biological applications of DNA-CNT hybrids have been proposed by many authors. For biological applications, most reactions of the hybrids with biomolecules are carried out in aqueous solutions. AFM imaging in liquids provides significant information about the morphology of the DNA-CNT hybrids in aqueous solutions.

## 3. Scanning Tunneling Microscopy Studies on DNA-CNT Hybrids

Although STM is one of the most popular types of SPMs, only a few studies have been published on DNA-CNT hybrids. One of the first studies (Iijima et al.) observed two types of DNA-MWNT hybrids by STM [50]. One type consisted of MWNT molecules wrapped with DNA molecules, while the other was a type of junction structure of DNA and MWNTs due to the insertion of DNA molecules into MWNTs. Scanning tunneling spectroscopy (STS) was also performed on the samples. Obtaining the current-voltage (*I*–*V*) curves at the nano-scale.

Wang et al. covalently attached ssDNA molecules at the end of SWNTs [51] and characterized the *I*–*V* curves of the conjugates by STS. In the study, the morphologies of ssDNA-SWNT hybrids were observed by scanning electron microscopy (SEM), transmission electron microscopy (TEM), and AFM. STM was used to measure the *I*–*V* curves. Using this approach, a biomimetic route of fabricating resonant tunneling diodes was found.

**Figure 3:**
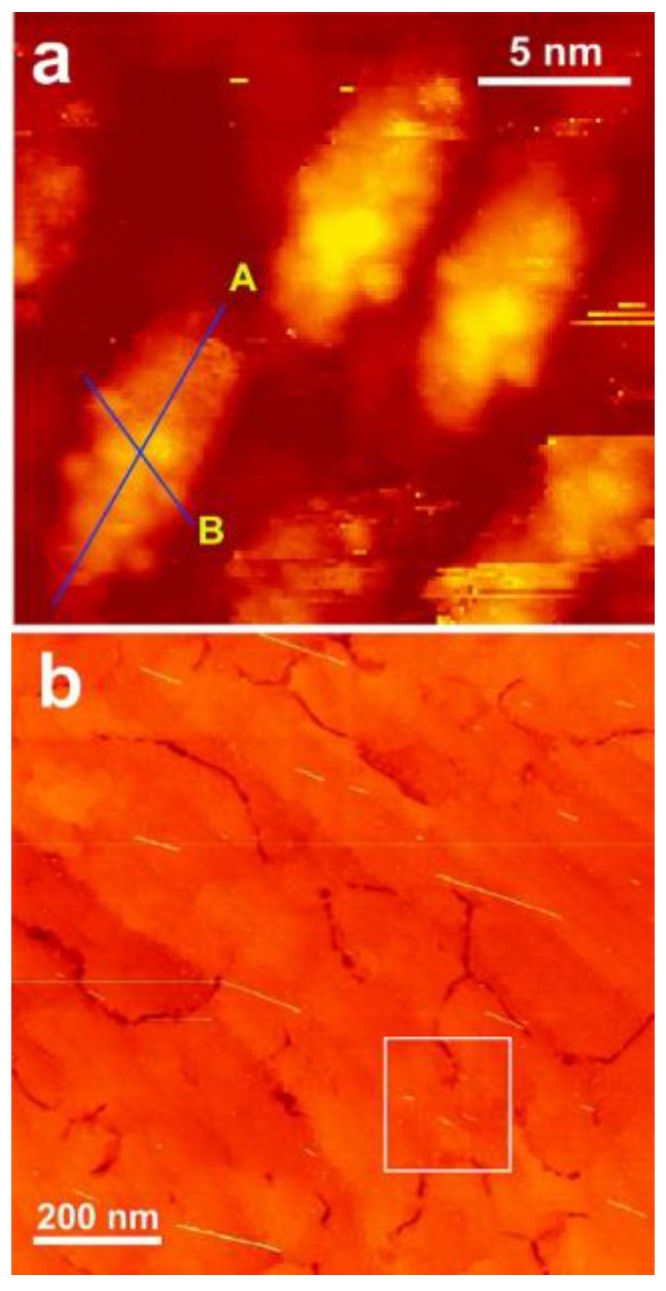
Scanning tunneling microscopy (STM) images of DNA-CNT hybrids deposited on p-doped Si(110) surface. (**a**) 21 nm × 21 nm scanning area; (**b**) 1 μm × 1 μm scanning area. For preparation of the DNA-CNT hybrids, 20 mer DNA (5′-NH_2_(C-6)GAGAAGAGAGCAGAAGGAGA-3′) was employed. Before the STM imaging, the sample was annealed at 550 °C for 30 min in vacuum (reprinted from reference [52] with permission).

Yarotski et al. published an article titled “Scanning Tunneling Microscopy of DNA-Wrapped Carbon Nanotubes”, where STM was used for the experimental characterization of DNA-CNTs. Theoretical simulation was also performed to explain the interaction between CNTs and DNA molecules [52]. Figure 3 shows STM images of DNA-CNT hybrids on Si (110). Furthermore, Kilina et al. discussed the stability criteria based on AFM measurements and theoretical calculations [53].

In the 1990s, STM studies on DNA molecules on flat conductive surfaces such as highly pyrolytic graphite (HOPG) were a trend in nanoscopic DNA research. However, HOPG was the “mimic for DNA” at the time, and most researchers employed AFM for DNA observation [54]. AFM, especially in intermittent contact mode, can be used to stably visualize the morphology of DNA molecules. Recently, several researchers succeeded in distinguishing the four DNA bases by STM in ultrahigh vacuum. As a substrate, Cu(111) was employed instead of HOPG or Au(111) [55,56,57].

Although STM imaging in ultrahigh vacuum is not a common method for biological researchers, STM has advantages in the examination of the electrical properties of DNA-CNT hybrids, as well as DNA molecules. Thus, STM has great potential as a valuable tool in the study of DNA-CNT hybrids.

## 4. Electrical Properties of DNA-CNT Hybrids Studied by Atomic Force Microscopy

There are several additional functions of AFM used to measure electrical properties of samples. One such option is Kelvin force microscopy (KPFM) [58,59,60,61]. Although it is not necessary for the AFM probe to be conductive for usual AFM imaging, a conductive probe is employed for KPFM. In many cases, the probe surface is coated with gold. A sample should be placed on a conductive substrate surface. Then, AC voltages are added between the probe and the sample. By monitoring the behaviors of the probe, the surface morphology and surface potential of the sample can be obtained. The surface potential is detected as the difference in work functions between the probe and the sample. The electrostatic force microscope (EFM) and conductive AFM (CAFM) are related to KPFM, but are slightly different microscopes [62,63,64,65,66,67,68].

There have been several studies on DNA and CNTs performed using KPFM, EFM, and CAFM. For example, Cui et al. demonstrated in situ imaging of SWNTs by AFM, KPFM, and EFM measurements for the same SWNT samples. It was found that the properties of the metal electrode/nanotube interface control the characteristics of field-effect transistors that were made with SWNTs.

Several studies have also been published in the area of DNA research. Ullien et al. presented CAFM images and *I*–*V* curves of dsDNA molecules on Au(111) surfaces [63,69]. Leung et al. succeeded in obtaining KPFM images of DNA molecules on insulating substrates [69]. For this purpose, dual-frequency mode was developed to minimize the tip-sample distance by modifying the commercially available SPM controller.

**Figure 4:**
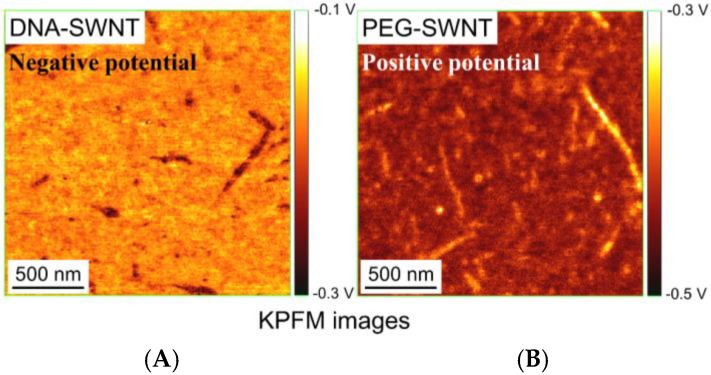
Kelvin force microscopy (KPFM) images of DNA-SWNT hybrids and SWNTs functionalized with PEG. (**A**) DNA-SWNT hybrids. Hybrids of thymine 30 (T30) were used as ssDNA; (**B**) polyethylene glycol (PEG)-SWNTs. Samples were deposited on conductive Au-Pd-coated mica surface. DNA-SWNT hybrids and PEG-SWNT revealed negative and positive surface potential against the Au-Pd surface, respectively (reprinted from reference [71] with permission).

As for DNA-CNT hybrids, several reports have been published. Lu et al. studied the low-frequency dielectric polarization of SWNTs by EFM [70], and found that metallic and semiconducting SWNTs showed significantly different dielectric responses. Although DNA was not discussed, it was used to disperse SWNTs before the EFM measurements. As the focus of the study was on SWNT characterization, the authors indicated that DNA was not necessary. In a study that focused on DNA molecules of DNA-CNT hybrids, Hayashida et al. revealed KPFM images of ssDNA-SWNT hybrids on Au(111) surfaces [71]. For comparison, SWNTs that were covalently decorated with PEG (PEG-SWNT) were also characterized [71]. As a result, DNA-SWNT and PEG-SWNT revealed positive and negative surface potentials, respectively (Figure 4). Zhu et al. reported a unique approach using CAFM [68]. They connected two SWNTs with *p*-phenylenediamine, and succeeded in measuring resistance of the junction. Although the hybrids were not DNA-SWNT hybrids, this approach may be useful for research on DNA-SWNT hybrids.

The characterization of the electrical properties of DNA-CNT hybrids is important in understanding their fundamental structures and functions. However, at this time, there are few studies that have focused on these aspects. It is possible that advanced AFM methods are not popular in biomaterials research. Future collaborations between biologists and physicists may achieve progress in this field.

## 5. Mechanics of DNA on Carbon Nanotubes

Force spectroscopy using AFM is one of the most specific and attractive approaches to study nanobiomaterials. First, a sample surface is observed by AFM imaging or other microscopic imaging methods. Then, the probe is stopped over the sample. To obtain force curves, it is necessary to let the sample or probe move up and down. By monitoring the bending of the cantilever, the force between the probe and the sample can be measured.

Single molecule force spectroscopy of DNA-CNT hybrids was demonstrated by Iliafar et al. in 2014 [72], with hybrids of SWNTs and four types of DNA tracts: poly(A), poly(C), poly(G), and poly(T). Then, single ssDNA molecules were peeled from SWNT surfaces. By force curve analysis, it was concluded that the order of binding free energy between ssDNA and SWNTs was poly(A) > poly(G) > poly(T) > poly(C) (38.1 ± 0.2; 33.9 ± 0.1; 23.3 ± 0.1; 17.1 ± 0.1 *k*_B_T, per nucleotide). Furthermore, the various interactions of DNA molecules adsorbed on some surfaces were compared.

Although there is only one published study that discusses the peeling force of DNA molecules from CNTs, there are several reports about that from graphite. Manohar et al. revealed the peeling of ssDNA molecules from graphite surfaces to obtain the binding energy between DNA and graphite [73]. It was found that the peeling forces for polythymine and polycytosine were 85.3 ± 4.7 pN and 60.8 ± 5.5 pN, respectively. These values were substantially independent of salt concentration and the rate of detachment. Wei et al. attached amino-functionalized ssDNA (5′-NH_2_-(CH_2_)_6_-AGT CAG TGT GGA AAA TCT CTA GC-3′) on an AFM probe [74]. In the first experiment, the ssDNA on the AFM probe was adsorbed on a graphite surface and then peeled from the surface. In the second experiment, a complementary ssDNA or a single mismatch complementary ssDNA was hybridized to the ssDNA on the AFM probe before peeling. It was found that the peeling force was decreased by almost 35.5% in the case of hybridized DNA molecules. The interaction between DNA and protein molecules was also measured by this approach. Recently, Wei et al. reported direct force measurements by peeling heteropolymer ssDNA from a graphite surface [75].

**Figure 5:**
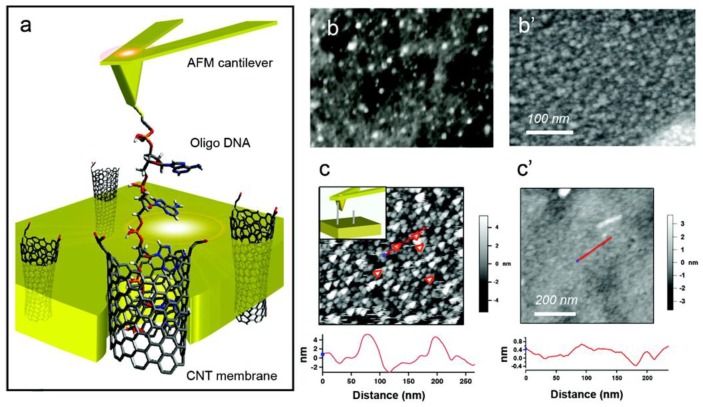
Frictionless sliding of ssDNA in CNT pore. (**a**) Schematic view. CNTs were vertically aligned in a rigid polymer matrix; (**b**) High-resolution scanning electron microscopy (SEM) image and (**c**) AFM image of the CNT membrane. (**b’**) SEM image and (**c’**) AFM image of control polymer film sample. Bottom cross-sections correspond to red lines on AFM images (reprinted from reference [76] with permission).

Also of interest is the ejection of DNA molecules from CNTs. Lulevich et al. inserted single ssDNA molecules attached to an AFM probe into CNT pores, and then ejected the ssDNA from the pore by pulling with the AFM probe [76]. As a result, constant force was observed during the pulling, and thus, it was concluded that nanotube pore walls are frictionless for DNA sliding. Figure 5 shows a schematic picture of DNA pulling from SWNT pores and several SEM and AFM images of the DNA-CNT sample.

Although the number of studies published is limited, force spectroscopy based on AFM is helping to elucidate unique knowledge on the interactions between DNA and nanocarbons, including CNTs. We predict that further experimentation with force spectroscopy and collaborations with theoretical approaches are likely.

## 6. Optical Properties of DNA-CNT Hybrids by Near-Field Scanning Optical Microscopy

Near-field scanning optical microscopy (NSOM) is a unique application within the SPM family [77,78,79]. Similar instruments are often referred to as scanning near-field optical microscopes (SNOM). In NSOM, a sample is located in an evanescent field; then, specific evanescent waves from the sample are detected. In some systems, an optical fiber is used as a light source or detector [80,81]. In the system referred to as the tip-enhanced near-field optical microscope (TENOM), a metallic AFM probe is used to produce an evanescent field [82,83,84]. Some researchers have used a prism as a substrate, and samples were deposited on the prism surface. In this scenario, the backside of the sample is irradiated with light [85,86].

NSOM has been used to study DNA molecules since the 1990s. For example, Garcia-Parajo et al. simultaneously obtained shear-force and near-field fluorescence images of DNA fragments with 1000 base pairs labeled with Rhodamine 6G (R6G), using an aperture-type NSOM [87]. At that time, the optical resolution of individual fluorophores was 70 nm at full-width at half-maximum. Several years later, Kim et al. observed denatured lambda DNA with YOYO-1 dyes as near-field fluorescence images and AFM topographical images [88,89]. Ma et al. achieved sub-10-nm resolution for fluorophores, and observed the helical rise of A-form DNA using an apertureless NSOM [90]. It is obvious that the improvement in the resolution of near-field fluorescence images has been one of the most important research targets in the NSOM field [91].

**Figure 6:**
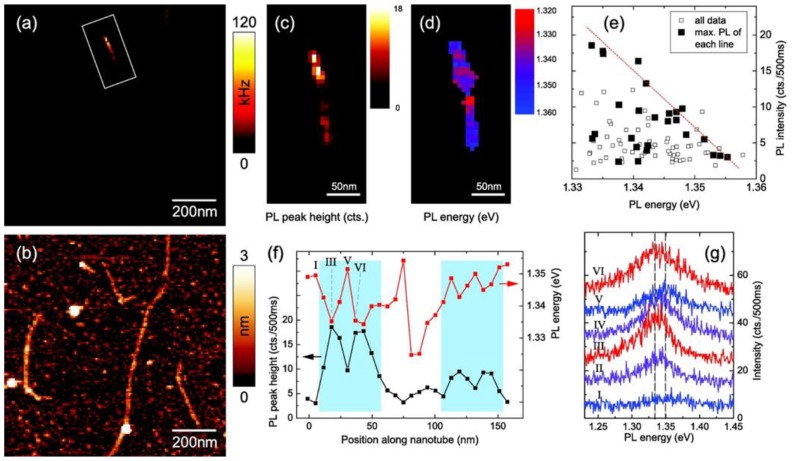
Exciton localization in a 160 nm long (9,1) SWNT. (**a**,**b**) Near-field photoluminescence (PL) image and corresponding topography showing several luminescent nanotubes; (**c**,**d**) Maps of the PL intensity and energy derived from a subsequent spectroscopic image of the area marked in panel (**a**) by peak fitting the recorded spectra at each image pixel; (**e**) Correlation between PL intensity and energy for all image pixels (open squares), with the brightest pixel of each image line being highlighted (filled squares) to account for the influence of finite spatial resolution (see text); (**f**) PL intensity and energy plotted along the nanotube; (**g**) Near-field spectra from six adjacent positions (*I*–*VI*) along the nanotube, equally spaced by 6.5 nm and marked in panel (**e**). The PL energy shifts by up to 15 meV (range indicated by the two dashed lines) between two neighboring positions (reprinted from reference [92] with permission).

Hartschuh’s group has studied DNA-CNT hybrids using TENOM with an inverted confocal microscope. For example, Georgi et al. characterized SWNTs wrapped with (GT)_10_ ssDNA [92] and succeeded in obtaining PL images of individual DNA-SWNT hybrids (Figure 6). The PL images revealed localization of excitons in semiconducting SWNTs at room temperature. In addition, it was suggested that when common surfactants such as sodium cholate were used, quantitative signal analysis was not possible, because of the formation of non-uniform films. Qian et al. simultaneously obtained near-field Raman, PL, and topographic images of DNA-SWNT hybrids [93,94]. In these studies, PL or Raman from SWNTs was investigated, and thus, DNA molecules were only used to disperse SWNTs not characterized on CNT surfaces.

In the NSOM studies on DNA molecules, DNA molecules were stained with dyes such as R6G and YOYO-1. The excitation wavelengths of these dyes are in the visible region; hence, samples were irradiated with visible lasers in the DNA studies. Contrarily, in SWNT studies, even though the SWNTs were wrapped with DNA molecules, the laser wavelengths were in the NIR range. DNA molecules were not stained when they were attached to SWNT surfaces.

It is expected that simultaneous evaluation of DNA-SWNT hybrids by NSOM with both visible and NIR lasers can be performed. Information on the interaction between DNA and SWNTs may be obtained by this approach. Recently, Ito et al. reported that PL spectra of SWNTs were fluctuated by the sequence of attached DNA molecules [95]. Their results include useful information for researchers using NSOM to study DNA-SWNT hybrids.

## 7. Fabrication of DNA-CNT Films Using an Atomic Force Microscopy Cantilever

Finally, a unique approach using SPM-related techniques is discussed. DNA-CNT hybrids have the potential to function as bio/chemical sensors. Paul et al. demonstrated an application of DNA-CNT hybrids for the selective detection of Hg(II) [96,97]. In this study, an ssDNA-CNT suspension was dropped between two Au electrodes using a micro-cantilever in order to make a thin film of hybrids between the electrodes. The maximum thickness of the film was 90 nm. Then, the I-V characteristics of the film were evaluated with Hg(II), Cd(II), and Pb(II) ions. As a result, the selective detection of Hg(II) using the DNA-CNT hybrid film was successful. In this work, SPM was not employed for measurement; however, thin films of DNA-CNT hybrids were fabricated with a micro-cantilever. 

## 8. Summary

After DNA-CNT hybrids were first fabricated in 2003, there has been nearly 15 years of research on their structures and physicochemical properties. Using this accumulation, many types of DNA-CNT hybrids can be prepared. Meanwhile, SPM has been utilized for almost 35 years. In addition to STM and AFM, many other nanoscopic characterization methods are available. Although there are few published studies, sophisticated nanoscopic research on DNA-CNT hybrids has been initiated by several researchers.
